# Improving of Sensitivity of PbS Quantum Dot Based SWIR Photodetector Using P3HT

**DOI:** 10.3390/ma14061488

**Published:** 2021-03-18

**Authors:** Kyeong-Ho Seo, Jaewon Jang, In Man Kang, Jin-Hyuk Bae

**Affiliations:** 1 School of Electronic and Electrical Engineering, Kyungpook National University, Daegu 41566, Korea; tjrudgh0826@naver.com (K.-H.S.); j1jang@knu.ac.kr (J.J.); imkang@ee.knu.ac.kr (I.M.K.); 2 School of Electronics Engineering, Kyungpook National University, Daegu 41566, Korea

**Keywords:** photosensitivity, poly(3-hexylthiophene-2,5-diyl), lead sulfide quantum dots, shortwave infrared photodetector, current on/off ratio

## Abstract

In this study, we improved the photosensitivity of the lead sulfide quantum dot (PbS QD)-based shortwave infrared (SWIR: 1.0–2.5 μm) photodetector by blending poly(3-hexylthiophene-2,5-diyl) (P3HT) with PbS QD. The PbS QD used for SWIR photoactive layer showed an absorption peak at 1410 nm. In addition, by using zinc oxide nanoparticles (ZnO NPs) as an interlayer, we obtained the stable current characteristics of our device. To confirm the effectiveness of P3HT on the PbS QD-based SWIR photodetector, we compared the electrical characteristics of a PbS QD-based device with a hybrid P3HT:PbS QD-based device. In the reverse bias region, the current on/off ratio of the PbS QD-based device was 1.3, whereas the on/off ratio of the hybrid P3HT:PbS QD-based device was 2.9; 2.2 times higher than the PbS QD-based device. At −1 V, the on/off ratio of the PbS QD-based device was 1.3 and the on/off ratio of the hybrid P3HT:PbS QD-based device was 3.4; 2.6 times higher than the PbS QD-based device. The fabricated P3HT:PbS QD-based device had the highest on/off ratio when −1 V voltage was applied.

## 1. Introduction

Shortwave infrared (SWIR: 1.0–2.5 μm) photodetectors are widely used in various fields such as telecom, temperature measurement, remote sensing, and spectroscopy [1,2,3,4]. In general, compound semiconductors, such as InGaAs, InAs, are applied to the fabrication of SWIR photodetectors. However, these materials require molecular beam epitaxial process to grow which have the high fabrication cost and difficulty of large-scale production [5,6]. To overcome these difficulties, many researchers have paid attention to lead sulfide quantum dots (PbS QDs), another SWIR active material, due to cost-effectiveness and large-scale manufacturability [7]. Furthermore, PbS QDs have the advantages of having high stability in air, size tunability, and flexible substrate compatibility [8,9,10,11], so this material emerges as a promising candidate for SWIR detection.

To date, the Schottky structure of indium tin oxide (ITO)/PbS QD/aluminum (Al) is one of the most commonly used structures [12,13]. However, the ITO/PbS QD/Al structure is reported to have poor performance due to bad rectification characteristics [14,15]. To overcome this drawback, zinc oxide nanoparticles (ZnO NPs) were used as an interlayer between PbS QDs and Al, preventing the Al diffusion and stabilizing the PbS QD layer [14,16]. ZnO NPs are one of the n-type semiconducting materials that have good transparency, wide bandgap of 3.37 eV, and good electron mobility characteristics [17,18]. Kwon et al. fabricated a PbS QD-based uncooled SWIR photodetector with stable current characteristics by using a ZnO NP interlayer.

Over the past few decades, PbS QDs have been used in optoelectronic devices along with conjugated polymers. A conjugated polymer is an organic material that has the advantage of flexibility, lightweight, low cost, and large-scale manufacturability [19,20]. Among the numerous conjugated polymers, poly(3-hexylthiophene-2,5-diyl) (P3HT) is one of the most widely used p-type semiconducting polymers [21]. In addition, it is relatively stable, has high hole mobility, and provides a shorter hole extraction time as a hole transport layer [22,23], contributing to improving the electrical conductivity of the electronic devices. A hybrid P3HT:PbS QD nanocomposite has the advantages of both P3HT and PbS QD and is still widely used in photodetectors and photovoltaics [24,25,26]. Xu et al. showed the operating mechanism of a hybrid P3HT:PbS QD-based device via energy level diagrams. In the literature, the highest occupied molecular orbital (HOMO) gap between PbS QD (−5.1 eV) and P3HT (−4.9 eV) is very narrow, enabling efficient hole transport. Such proper use of these donor-acceptor materials can expect the optimal photogenerated charge separation, contributing to improving the photosensitivity of the electronic devices [25,27].

Based on these backgrounds, this study focused on the photosensitivity improvement of the PbS QD-based SWIR photodetector with or without P3HT. A blending of P3HT and PbS QD contributed to the improvement of electrical characteristics by providing the optimal photogenerated charge separation condition. The photosensitivity evaluation was specifically quantified through the current on/off ratio. These results showed that the use of an optimized donor/acceptor had a positive effect on the performance of the PbS QD-based SWIR photodetector.

## 2. Materials and Methods

### 2.1. Fabrication of the Hybrid P3HT:PbS QD Solution

**Figure 1:**
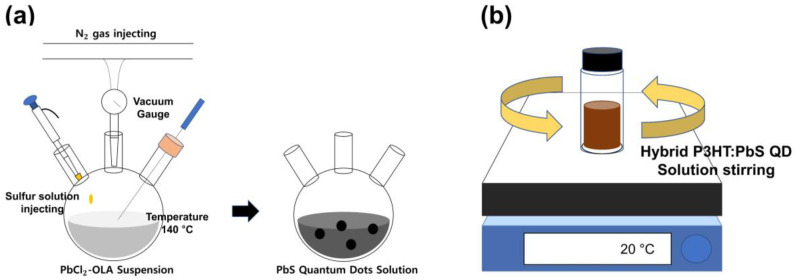
(**a**) Schematic diagram of PbS quantum dots synthesis process and (**b**) the fabrication process of hybrid P3HT:PbS QD solution.

Prior to preparing the hybrid P3HT:PbS QD solution, we synthesized the PbS quantum dots. A 1 mmol amount of lead chloride (PbCl_2,_ 99.999%, Sigma-Aldrich, St. Louis, MO, USA) was dissolved in 5 mL of oleylamine (OLA, 70%, Sigma-Aldrich, St. Louis, MO, USA) under N_2_ conditions for 30 min at room temperature. Then, the dissolved solution was heated to 160 °C for 1 h. The heated solution was then cooled to 120 °C for 15 min. During the cooling process, a three-neck flask was sealed to hold the vacuum condition. After 15 min, the three-neck flask was opened, and N_2_ gas was reinjected. While the PbCl_2_-OLA suspension was prepared, 0.36 mmol of sulfur (S, 99.998%, Sigma-Aldrich, St. Louis, MO, USA) was dissolved in 1 mL of OLA at room temperature under N_2_ conditions. The prepared sulfur stock solution was quickly injected into the PbCl_2_-OLA suspension under stirring, and the stirring continued for an additional 30 min at 150℃. The resultant was rinsed with ethyl alcohol (EtOH, 99.9%, Samchun Pure Chemical CO., Ltd., Seoul, Korea), then centrifuged, precipitated, and dispersed with toluene (99.8%, Sigma-Aldrich, St. Louis, MO, USA) at a concentration ratio of 30 mg/mL. Based on the synthesized PbS QDs, we fabricated the hybrid P3HT:PbS QD solution. P3HT (5 mg) was dissolved in the 1 mL of PbS quantum dots for 1 h on a hot plate at 20 °C, and the color of the solution changed from black to maroon. Figure 1 shows the fabrication process of the hybrid P3HT:PbS QD solution.

### 2.2. Synthesis of ZnO NPs

**Figure 2:**
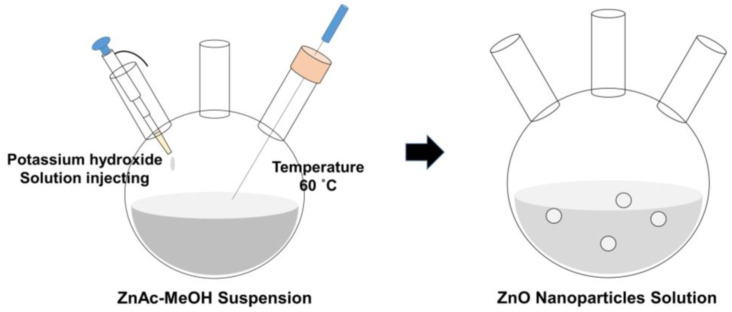
Schematic diagram of ZnO nanoparticle synthesis process.

A 2.46 g amount of zinc acetate dihydrate (Zn(CH_3_COO)_2_ 2H_2_O, 98%, Sigma-Aldrich, USA) was dissolved in 110 mL of methyl alcohol (MeOH, 99.9%, Duksan Pharmaceutical CO., Ltd., Seoul, Korea) and 1.152 g of potassium hydroxide (KOH, 90%, Sigma-Aldrich, USA) was dissolved in 60 mL of MeOH at 60 °C for 1 h. The dissolved KOH solution was injected into the ZnAc solution at a rate of (1 mL/s) and dissolved for 1 h. The resultant was rinsed with isopropyl alcohol (IPA, 99.9%, Duksan Pharmaceutical CO., Ltd., Korea) and hexane (95%, Duksan Pharmaceutical CO., Ltd., Korea) mixed together and then stored for 24 h in a 5 °C refrigerated reagent cabinet. The precipitate was centrifuged at 3000 rpm and dispersed with ethyl alcohol (EtOH, 99.9%, Samchun Pure Chemical CO., Ltd., Seoul, Korea) at 30 mg/mL of concentration ratio. Figure 2 shows a schematic diagram of the synthesis process of ZnO nanoparticles.

### 2.3. Fabrication of the SWIR Photodetector

**Figure 3:**
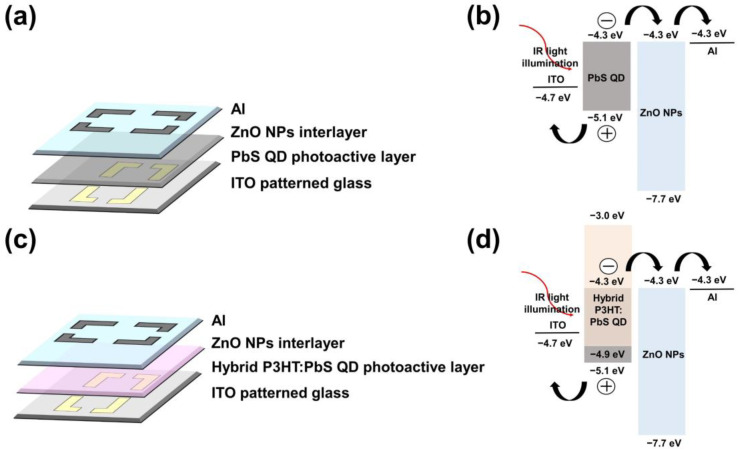
Schematic diagram of fabricated devices: (**a**) The structure of the PbS QD-based device, (**b**) the bandgap of the P3HT:PbS QD-based device, (**c**) the structure of the hybrid P3HT:PbS QD-based device and (**d**) the bandgap of the hybrid P3HT:PbS QD-based device.

All devices were fabricated through the spin-coating method. The anode was ITO, a transparent electrode, which was patterned on a 30 mm × 30 mm glass substrate. The glass substrate was rinsed with acetone (99.9%, Duksan Pharmaceutical CO., Ltd., Ansan, Korea), methanol, and isopropyl alcohol in an ultrasonic cleaner (NXPC-2010, Kodo Technical Research Co., Ltd., Gyeonggi-do, Korea) for 10 min sequentially to remove organic residues and impurities. To remove the residual solvent, the rinsed glass was dried under N_2_ gas and heat-treated on a hot plate at 150 °C for 5 min. A UV ozone treatment was performed for 20 min to make the substrate hydrophilic by controlling the surface energy. Both photoactive layers, the pure PbS QD solution, and the hybrid P3HT:PbS QD solution were dispensed on the ITO glass substrate and spin-coated at 3000 rpm, respectively. Both spin-coated photoactive layers were annealed for 30 min in a vacuum oven at 110 °C for activation. Then, the ZnO NP layer used as an electron transport layer was dispensed on the formed photoactive layer, spin-coated at 1500 rpm, and then annealed at 90 °C for 30 min. Finally, aluminum was deposited as a cathode of the device to a thickness of 150 nm by thermal evaporation. In the completed device, the anode and cathode cross-contact area were 9 mm^2^ where the device is activated. Figure 3 shows the schematic diagram of the fabricated devices, Figure 3a shows the structure of the fabricated PbS QD-based structure, and Figure 3b shows the bandgap of the fabricated PbS QD-based device. When light irradiates the PbS QD-based device, electron-hole pairs (EHP) are generated within the device, then the generated EHPs are extracted to both electrodes while the external electric field was applied to the device. Figure 3c shows the structure of the fabricated hybrid P3HT:PbS QD-based device and Figure 3d shows the bandgap structure of the fabricated P3HT:PbS QD-based device. Unlike Figure 3b, PbS QD was blended with P3HT, so the bandgap of P3HT overlapped with PbS QD. By overlapping the bandgap, holes generated by light in the PbS QD can be quickly extracted to the ITO electrode through the P3HT [24,28].

### 2.4. Measurements

The characteristics of the synthesized PbS QD and ZnO NPs were measured using a UV-VIS-NIR spectrometer (Cary 5000, Agilent Tech., Santa Clara, CA, USA). We have been able to confirm the properties of the synthesized nanoparticles through the absorption spectrum. For the synthesized PbS QD, about 80% of toluene was injected into the cuvette to set a reference for the sample. For the synthesized ZnO NPs, about 80% of the synthesized ZnO NPs were injected. The characteristics of the fabricated photodetectors were confirmed by I-V characteristics. All fabricated devices were measured in the darkroom to block external light. The IR light source was Deuterium-Halogen Light Source (SL5, StellarNet, Inc., Tampa, FL, USA). The light intensity was 1 W/cm^2^, which provided a 2500 nm wavelength. To measure the I-V characteristics of the fabricated photodetector, we used the M6100 OLED I-V-L Test System (McScience, Yeongtong, Suwon, Korea) and the source meter (Keithley 2400, Tektronix, Inc., Beaverton, OR, USA).

## 3. Result and Discussion

**Figure 4:**
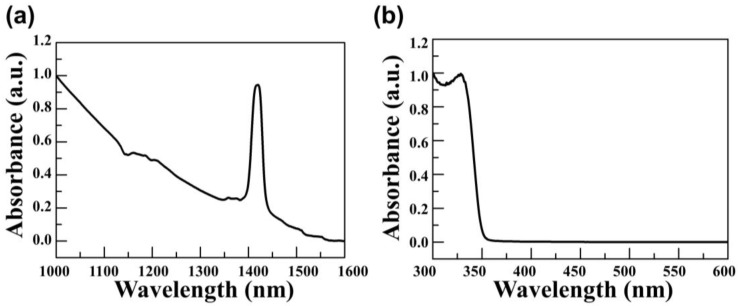
Absorbance spectrum of synthesized nanoparticles: (**a**) PbS quantum dot and (**b**) ZnO nanoparticles.

The characteristics of the synthesized PbS QD and ZnO NPs were confirmed using a UV-VIS-NIR spectrometer. Figure 4a shows the absorption spectrum of the synthesized PbS QD; it exhibited an absorption spectrum wavelength of 1600 nm. At 1410 nm wavelength, an absorption peak appeared. This means that our synthesized PbS QD absorbed 1410 nm of light, which is the SWIR region. When the photons struck the semiconductor, the electrons in the valence band excite into the conduction band and then formed the excitons. These excitons underwent a charge separation process and contributed to the improvement of the photoconductivity of the device.

ZnO NPs are wide bandgap of 3.37 eV metal oxides that absorb ultraviolet (UV) light. Although these materials do not absorb IR light, absorbance measurements were performed to confirm our ZnO NPs were successfully synthesized. Figure 4b shows the absorption spectrum of synthesized ZnO NPs. The synthesized ZnO NPs exhibited an absorption spectrum wavelength of 600 nm. The absorption peak appeared at 325 nm wavelength. It means that our synthesized ZnO NPs absorbed 325 nm of light, and then exciton formed. However, these excitons generated by ultraviolet light were not related to the photoconductivity of the device. As our synthesized ZnO NPs showed a light absorption peak at 325 nm, it was confirmed that there were no functional problems in the synthesized ZnO NPs. Although Figure 4b did not show the parameter of electron transport, it showed that ZnO NPs were properly synthesized, so our synthesized ZnO NPs were used as a device interlayer.

**Figure 5:**
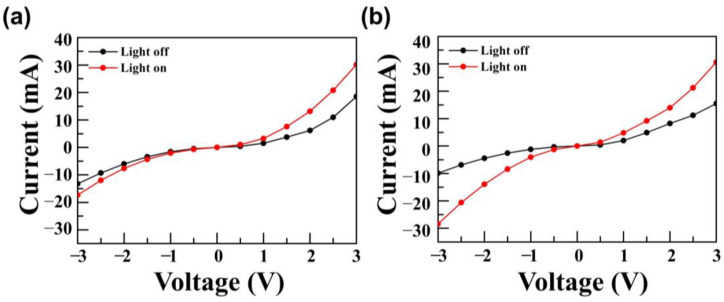
I-V characteristics of fabricated SWIR photodetector: (**a**) PbS QD-based device and (**b**) hybrid P3HT:PbS QD-based device.

Based on these synthesized PbS QD and ZnO NPs, a SWIR photodetector was fabricated and confirmed the electrical performance of devices with or without P3HT. The I-V characteristics of each fabricated devices with or without light illumination were measured and the current on/off ratio was calculated to determine the current change of the fabricated devices. “Light off” means the current when the device was not illuminated with IR light, while “Light on” means the current when the device was illuminated with IR light. By dividing the on current by the off current, we confirmed the change ratio of both currents. The voltage was applied from −3 V to 3 V, and the step voltage was set to 0.5 V. Figure 5 shows the I-V characteristics of the fabricated devices.

In the reverse bias region, for the PbS QD-based device without P3HT, the dark current was −13.3 mA, the light current was −17.6 mA, and the current on/off ratio was 1.3. On the other hand, for the hybrid P3HT:PbS QD-based device, the dark current was −9.9 mA, the light current was −28.5 mA, and the current on/off ratio was 2.9. Notably, the current on/off ratio of the hybrid P3HT:PbS QD-based device was about 2.2 times higher than the current on/off ratio of the PbS QD-based device [25,29]. The P3HT was considered to improve the conductivity of the photogenerated charge from PbS QD, resulting from a higher light current of the fabricated PbS QD-based SWIR photodetector. Moreover, by blending P3HT and PbS QD, the photogenerated charge separation was more efficient than PbS QD only device, resulting in the improvement of the photosensitivity [27]. The HOMO level of P3HT (−4.9 eV) adjacent to the PbS QD (−5.1 eV) provided optimal conditions for this charge separation [24]. Moreover, the current on/off ratio was the highest in which the applied voltage was −1 V. In the PbS QD-based device, the dark current was −1.6 mA, the light current was −2.1 mA, and the current on/off ratio was 1.3. In the P3HT:PbS QD-based device, the dark current was −1.2 mA, the light current was −4.1 mA, and the current on/off ratio was 3.4. The results obtained from the devices are shown in Table 1.

**Figure 6:**
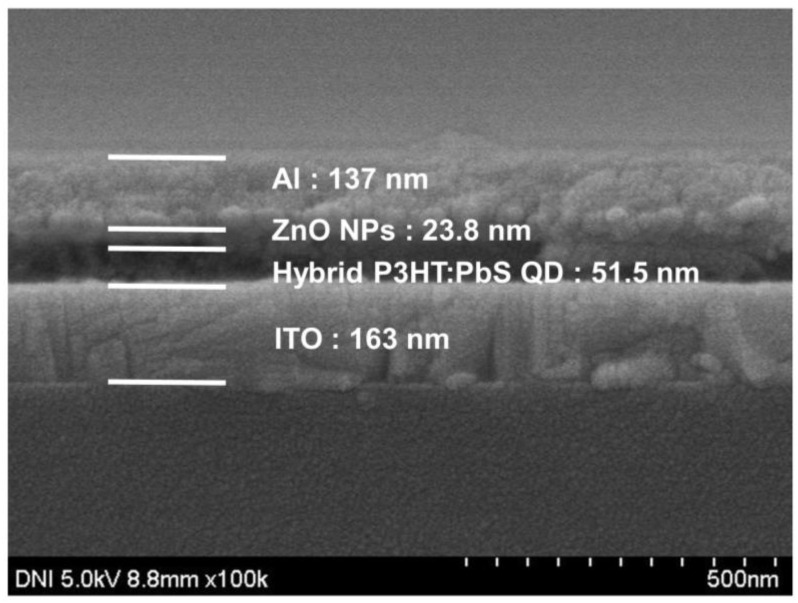
FE-SEM image of fabricated hybrid P3HT:PbS QD-based device.

The structure of the fabricated hybrid P3HT:PbS QD-based device was shown through the FE-SEM image. Figure 6 shows the FE-SEM image of the fabricated hybrid P3HT:PbS QD-based device. The ITO electrode was 163 nm, the hybrid P3HT:PbS QD layer was 51.5 nm, the ZnO NP layer was 23.8 nm and the Al electrode was 137 nm. Since orthogonal solvents were used in the device fabrication process, as shown in Figure 4, each layer could be deposited independently.

## 4. Conclusions

In this study, we improved the photosensitivity of the SWIR photodetector based on PbS QD by using P3HT. To detect SWIR, we used a synthesized PbS QD with an excitonic peak at 1410 nm. Additionally, we used ZnO NPs as an interlayer to maintain stable current characteristics. In the reverse bias region, for the PbS QD-based device without P3HT, the dark current was −13.3 mA, the light current was −17.6 mA, and the current on/off ratio was 1.3. For the hybrid P3HT:PbS QD-based device, the dark current was −9.9 mA, the light current was −28.5 mA, and the current on/off ratio was 2.9. The current on/off ratio of the hybrid P3HT:PbS QD-based device was 2.2 times higher than the PbS QD-based device. At −1 V, the current on/off ratio of the PbS QD-based device was 1.3, and the hybrid P3HT:PbS QD was 3.4, the highest among the measured currents. These results will contribute toward developing the next-generation electronic device industry by obtaining SWIR optoelectronic device performance enhancement technology.

## Figures and Tables

**Table 1:** The results of the measured devices.

Properties	PbS QD From −3 V to −1 V	Hybrid P3HT:PbS QD From −3 V to −1 V
Dark current (mA)	From −13.3 to −1.6	From −9.9 to −1.2
Light current (mA)	From −17.6 to −2.1	From −28.5 to −4.1
On/off ratio	From 1.3 to 1.3	From 2.9 to 3.4

## Data Availability

The data presented in this study are available on request from the corresponding author.
